# Glucagon-Like Peptide-1 Receptor Agonist Switching and Treatment Persistence in Adults Without Diabetes

**DOI:** 10.1001/jamanetworkopen.2026.1272

**Published:** 2026-03-10

**Authors:** Luyu Xie, Diego Anazco, Azucena Herrera Chancay, M. Sunil Mathew, Jackson M. Francis, Jaime P. Almandoz, Sarah E. Messiah

**Affiliations:** 1 Quantitative Biomedical Research Center, Department of Health Data Science and Biostatistics, Peter O’Donnell Jr School of Public Health, The University of Texas Southwestern Medical Center, Dallas; 2 Department of Internal Medicine, Division of Endocrinology, University of Texas Southwestern Medical Center, Dallas; 3 Department of Epidemiology, Peter O’Donnell Jr School of Public Health, University of Texas Southwestern Medical Center, Dallas; 4 Department of Pediatrics, University of Texas Southwestern Medical Center, Dallas; 5 Children’s Health System of Texas, Dallas

## Abstract

This cohort study evaluates glucagon-like peptide-1 (GLP-1) receptor agonist switching patterns and 12-month adherence and persistence among adults with overweight or obesity without diabetes.

## Introduction

Glucagon-like peptide-1 receptor agonists (GLP-1RAs) are increasingly used for obesity, but long-term adherence is suboptimal.^1,2,3^ Switching between agents is common, yet clinical practice patterns are not well described.^4^ This cohort study examined switching patterns and 12-month adherence and persistence among adults with overweight or obesity without diabetes.

## Methods

We conducted a retrospective cohort study using the Merative MarketScan Commercial Claims and Encounters Database from 2019 to 2024, which includes deidentified claims from approximately 40 million insured US individuals annually. The study was exempt from institutional review board approval due to use of deidentified data and followed Strengthening the Reporting of Observational Studies in Epidemiology (STROBE) reporting guidelines. Adults with overweight or obesity initiating GLP-1RA therapy were included if continuously enrolled for 12 months before and after initiation without prior GLP-1RA use since 2019 or a diabetes diagnosis in the preceding year (eFigure in Supplement 1). The first GLP-1RA fill defined the index date; patients were classified as switchers (changed agents within 12 months) or nonswitchers (remained on the index agent). We reported 12-month persistence (continuous therapy with ≤60-day gaps) and adherence (proportion of days covered [PDC]).^5^ Optimal adherence was defined as a PDC of 80% or more.^5^ A Sankey diagram visualized switching pathways. A 2-sided *P* < .05 defined statistical significance. Analyses were conducted using SAS version 9.4 (SAS Institute). Additional details and definitions appear in the eMethods in Supplement 1.

## Results

The study included 126 984 patients initiating GLP-1RA therapy (mean [SD] age, 45.1 [10.2] years; 102 047 [80.4%] female; 69 190 [54.5%] with class I-II obesity, 43 131 [34.0%] with class III obesity, and 14 663 [11.5%] with overweight) (Table). A total of 26 197 patients (20.6%) followed a switcher trajectory, transitioning between agents within the 12-month follow-up. Switchers demonstrated higher baseline rates of depression, sleep apnea, gastroesophageal reflux, asthma, and metabolic dysfunction–associated steatotic liver disease.

**Table.** Patient Characteristics and 12-Month Outcome After Glucagon-Like Peptide-1 Receptor Agonist (GLP-1RA) Initiation by Treatment Pattern, 2019 to 2024

Characteristic	Patients, No. (%)			*P* value^c^
	Total (N = 126 984)	Switchers (n = 26 197)^a^	Nonswitchers (n = 100 787)^b^	
Age at index, mean (SD)	45.1 (10.2)	44.9 (10.0)	45.1 (10.2)	.01
Sex				
Female	102 047 (80.4)	21 606 (82.5)	80 441 (79.8)	<.001
Male	24 937 (19.6)	4591 (17.5)	20 346 (20.2)	
BMI group^d^^,^^e^				
Overweight	14 663 (11.5)	2590 (9.9)	12 073 (12.0)	<.001
Obesity class 1 and 2	69 190 (54.5)	14 629 (55.8)	54 561 (54.1)	
Obesity class 3	43 131 (34.0)	8978 (34.3)	34 153 (33.9)	
Comorbidities^f^				
Hypertension	49 320 (38.8)	10 048 (38.4)	39 272 (39.0)	.07
Dyslipidemia	47 898 (37.7)	9789 (37.4)	38 109 (37.8)	.19
Depression	27 333 (21.5)	5976 (22.8)	21 357 (21.2)	<.001
Sleep apnea	23 028 (18.1)	4914 (18.8)	18 114 (18.0)	.003
Gastroesophageal reflux disease	21 772 (17.2)	4681 (17.9)	17 091 (17.0)	<.001
Asthma	12 130 (9.5)	2591 (9.9)	9539 (9.5)	.04
Metabolic dysfunction–associated steatotic liver disease	6425 (5.1)	1389 (5.3)	5036 (5.0)	.04
Coronary artery disease	3610 (2.8)	758 (2.9)	2852 (2.8)	.58
Index GLP-1RA prescription				
Index y				
2019	702 (0.5)	36 (0.1)	666 (0.7)	<.001
2020	9075 (7.1)	622 (2.4)	8453 (8.4)	
2021	18 630 (14.7)	2830 (10.8)	15 800 (15.7)	
2022	43 460 (34.2)	11 562 (44.1)	31 898 (31.7)	
2023	55 117 (43.4)	11 147 (42.6)	43 970 (43.6)	
Index drug				
Diabetes-indicated				<.001
Exenatide (Byetta)	89 (0.1)	17 (0.1)	72 (0.1)	
Exenatide (Bydureon)	17 (>.01)	2 (>.01)	2 (>.01)	
Liraglutide (Victoza)	1645 (1.3)	418 (1.6)	1227 (1.2)	
Dulaglutide (Trulicity)	4438 (3.5)	973 (3.7)	3465 (3.4)	
Semaglutide (Ozempic)	34 828 (27.4)	6875 (26.2)	27 953 (27.7)	
Semaglutide (Rybelsus)	4619 (3.6)	910 (3.5)	3709 (3.7)	
Tirzepatide (Mounjaro)	9866 (7.8)	1601 (6.1)	8265 (8.2)	
Obesity-indicated				
Liraglutide (Saxenda)	26 384 (20.8)	7664 (29.3)	18 720 (18.6)	
Semaglutide (Wegovy)	45 098 (35.5)	7737 (29.5)	37 361 (37.1)	
Tirzepatide (Zepbound)	0	0	0	NA
Utilization outcome				
12-mo persistence^g^	31 134 (24.5)	9537 (36.4)	21 597 (21.4)	<.001
Adherence (PDC), mean (SD)^h^	54.3 (30.3)	63.0 (24.6)	52.0 (31.2)	<.001
Adherent (PDC ≥80%)^h^	37 603 (29.6)	8729 (33.3)	28 874 (28.6)	<.001

Abbreviation: BMI, body mass index; NA, not applicable; PDC, proportion of days covered.

^a^ Patients who changed to a different GLP-1RA at any point during 12 months postinitiation.

^b^ Patients who remained on their index GLP-1RA during 12 months postinitiation regardless of treatment gaps.

^c^ χ^2^ analysis for categorical variables and *t* test for continuous variables.

^d^ Most recent diagnosis before the index date.

^e^ BMI categories were derived from the *International Statistical Classification of Diseases and Related Health Problems, Tenth Revision *codes.

^f^ Twelve months before index date.

^g^ Continuous therapy (any GLP-1RA) for 12 months postinitiation with gaps 60 days or less.

^h^ Days with medication supply divided by total follow-up days.

Overall, 12-month persistence across the entire cohort was 24.5% (31 134 patients). Switchers showed higher persistence (9537 switchers [36.4%] vs 21 597 nonswitchers [21.4%]; *P* < .001) and adherence (mean [SD] PDC, 63.0% [24.6] vs 52.0% [31.2]; *P* < .001). Optimal adherence occurred in 33.3% (8729 patients) of switchers vs 28.6% (28 874 patients) of nonswitchers (*P* < .001). Semaglutide formulations were the most common index agents, followed by liraglutide (Table).

As illustrated in the Sankey diagram (Figure), 100 787 patients (79.4%) remained on their initial GLP-1RA without switching during follow-up. Among the 26 197 (20.6%) who switched therapies, the most common initial transition was from liraglutide to semaglutide (6393 patients [24.4%]). While the vast majority of switchers (24 280 patients [92.7%]) changed medications only once, a small minority (1917 patients [7.3%]) underwent multiple switches, such as the sequence from liraglutide to semaglutide and then to tirzepatide. Injectable semaglutide formulations served as both common starting medications and frequent switch destinations, suggesting their central role in obesity care.

**Figure.**
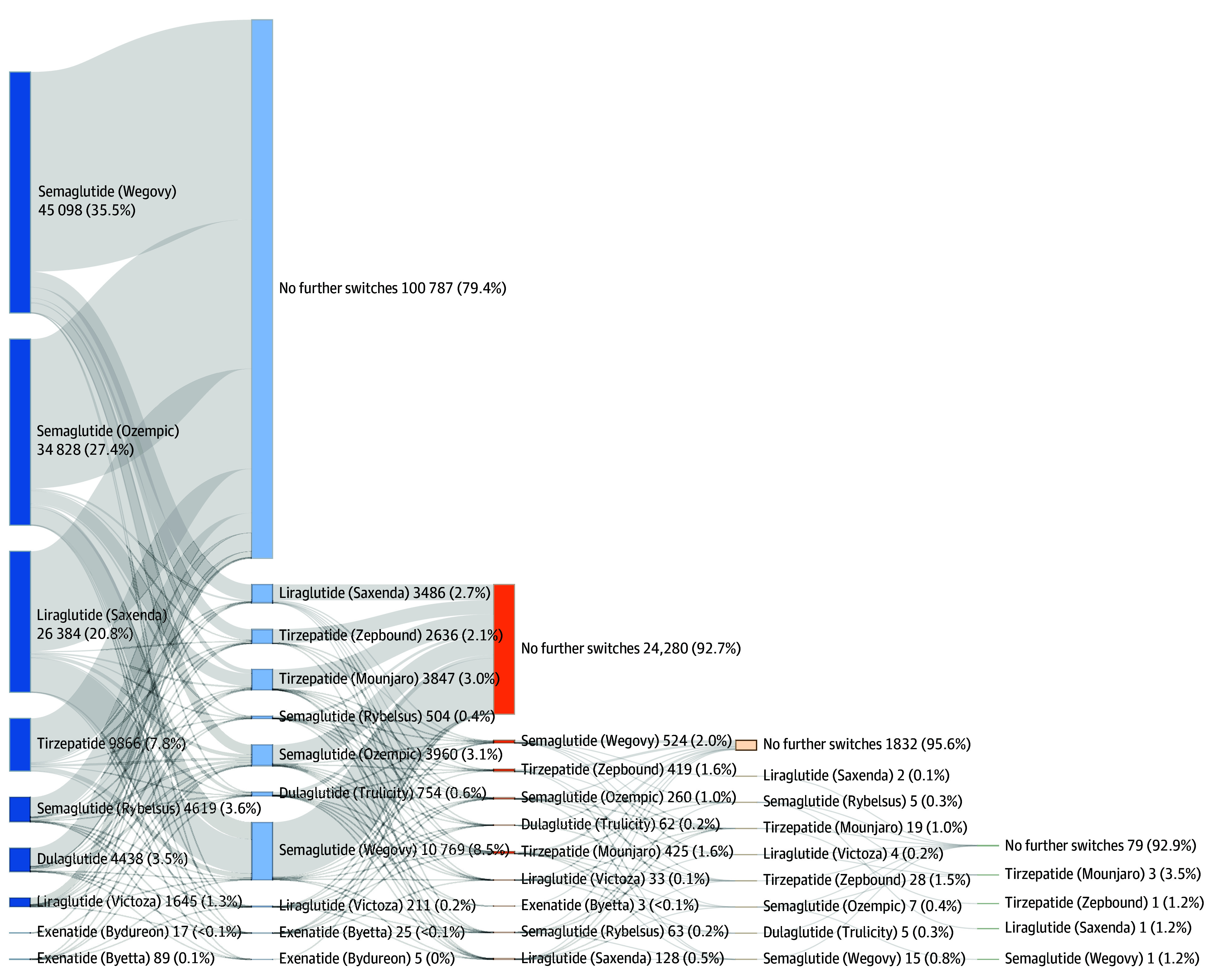
Sankey Plot of Glucagon-Like Peptide-1 Receptor Agonist (GLP-1RA) Switching Patterns (126 984 patients) GLP-1RA switching patterns among patients with overweight or obesity but without diabetes 12 months postinitiation. The diagram shows the flow of patients from initial medications (left) to subsequent treatment (right). Line thickness is proportional to the percentage of patients making each switch. Percentages in parentheses represent the proportion of patients from each starting medication who switched to the indicated treatment or remained on therapy without further switches.

## Discussion

In this large cohort of adults with overweight or obesity without diabetes, fewer than 1 in 4 patients remained on any GLP-1RA after 12 months. Switching between GLP-1RA agents was common and may reflect active therapy management rather than nonengagement, particularly as new formulations and weight management agents emerge.^4,6^

The rapid uptake of semaglutide and tirzepatide since 2021 illustrates how innovation is associated with dynamic treatment patterns in obesity care. These findings support reframing switching as part of long-term optimization rather than discontinuation. Clinicians should encourage continuity across switches and address coverage, cost, and adverse-effect barriers that limit persistence. Future studies should examine agent-specific and temporal differences in switching and persistence, with particular attention to dosing frequency, tolerability, access, and socioeconomic, geographic, and patient-reported factors that may influence long-term use.

This study has limitations. Comparisons between switchers and nonswitchers are subject to selection bias and immortal time bias; therefore, the findings should be interpreted descriptively rather than causally. The requirement for continuous enrollment may also introduce selection bias, and claims data do not capture out-of-pocket GLP-1RA use, which may lead to underestimation of treatment persistence and adherence.

In conclusion, persistence with GLP-1RA therapy remains low, and switching appears to be a common treatment trajectory among patients engaged in care. Interpreting switching as part of ongoing therapy may inform patient-centered obesity care.

## Notes

Supplement 1. eMethods. eFigure. Cohort Selection Steps

Supplement 2. Data Sharing Statement
