# Supplementary material for: Glucagon-Like Peptide-1 Receptor Agonist Switching and Treatment Persistence in Adults Without Diabetes
Source: JAMA Netw Open. 2026 Mar 10;9(3):e261272. doi: 10.1001/jamanetworkopen.2026.1272 (PMC12976791; doi:10.1001/jamanetworkopen.2026.1272)
Supplement: Supplement 1. — eMethods. eFigure. Cohort Selection Steps [file jamanetwopen-e261272-s001.pdf]

Supplemental Online Content

Xie L, Anazco D, Chancay AH, et al. GLP-1 receptor agonist switching and treatment persistence in adults without diabetes. *JAMA Netw Open*. 2026;9(3):e261272.
doi:10.1001/jamanetworkopen.2026.1272

eMethods.

eFigure. Cohort Selection Steps

This supplemental material has been provided by the authors to give readers additional information about their work.

© 2026 Xie L et al. JAMA Network Open.

eMethods

Study Design and Data Source

We conducted a retrospective cohort study using the Merative MarketScan Commercial Claims and Encounters Database from 2019-2024. This database contains de-identified healthcare claims from employer-sponsored health plans covering approximately 40 million individuals annually across the United States. This study was deemed exempt from institutional review board (IRB) approval as it utilized de-identified administrative claims data with no direct patient contact and followed the Strengthening the Reporting of Observational Studies in Epidemiology (STROBE) guidelines for reporting observational studies.

Study Population

We identified 126,984 adult patients with overweight or obesity who initiated GLP-1RA therapy between 2019-2024 (defined as no previous use before 2019), had continuous enrollment for 12 months pre- and post-initiation, and no diabetes diagnosis in the 12 months prior to initiation. (Supplemental Figure 1) The index date was defined as the first GLP-1RA prescription fill.

Treatment Pattern Classification

Patients were classified based on the following 12-month treatment patterns: (1) Switchers: changed to a different GLP-1RA during follow-up; and (2) non-switchers: remained on their index GLP-1RA throughout follow-up regardless of treatment gaps.

Outcome Measures

Primary outcomes included 12-month persistence and adherence. Persistence was defined as continuous therapy with any GLP-1RA for 12 months post-initiation, allowing gaps $\leq$60 days between fills at the class level. Adherence was measured using proportion of days covered (PDC), calculated at the

© 2026 Xie L et al. JAMA Network Open.

GLP-1RA class level as the number of unique days within the 365-day post-index period covered by any GLP-1RA supply divided by 365 days. Adherence was based on pharmacy fill dates and days' supply, hospitalization days were not excluded from the denominator, and PDC ⩾80% defined optimal adherence.

Covariates

Patient characteristics included age, sex, body mass index (BMI) categories (overweight [25.0-29.9 kg/m²], obesity class I, II [30.0-39.9 kg/m²], obesity class III [⩾40 kg/m²]) using the most recent diagnosis before the index date, and comorbidities identified using ICD-10 codes during the 12 months pre-index.

Statistical Analysis

Patient characteristics were compared between treatment groups using chi-square tests for categorical variables and t-tests for continuous variables. Statistical significance was set at $P<0.05$. All analyses were conducted using SAS version 9.4. Sankey plot was created using the "networkD3" package in R version 4.4.1.

© 2026 Xie L et al. JAMA Network Open.

eFigure. Cohort Selection Steps

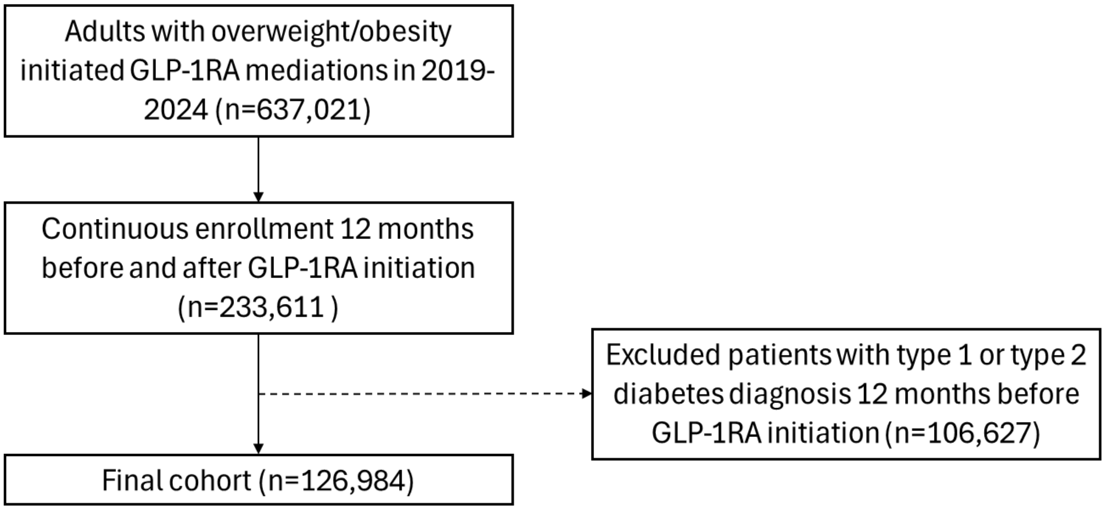

© 2026 Xie L et al. JAMA Network Open.
