# Supplementary material for: Glucagon-Like Peptide-1 Receptor Agonist Switching and Treatment Persistence in Adults Without Diabetes
Source: JAMA Netw Open. 2026 Mar 10;9(3):e261272. doi: 10.1001/jamanetworkopen.2026.1272 (PMC12976791; doi:10.1001/jamanetworkopen.2026.1272)
Supplement: Supplement 2. — Data Sharing Statement [file jamanetwopen-e261272-s002.pdf]

Data Sharing Statement

Xie. Glucagon-like peptide-1 Receptor Agonist Switching and Treatment Persistence in Adults Without Diabetes. *JAMA Netw Open*. Published March 10, 2026.
doi:10.1001/jamanetworkopen.2026.1272

Data
Data available: No
